# Supplementary material for: HIVprotI: an integrated web based platform for prediction and design of HIV proteins inhibitors
Source: J Cheminform. 2018 Mar 9;10:12. doi: 10.1186/s13321-018-0266-y (PMC5845081; doi:10.1186/s13321-018-0266-y)
Supplement: Supplementary file 2 — Additional file 2. Source code of HIVProtI web server. [file 13321_2018_266_MOESM2_ESM.zip › HIVprotI_Source-code/hivprotI/weka-3-6-6/doc/weka/classifiers/rules/part/ClassifierDecList.html]

ClassifierDecList


---


|  |  |  |
| --- | --- | --- |
| **Overview** \| **Package** \| **Class** \| **Tree** \| **Deprecated** \| **Index** \| **Help** \| **Weka's home** | |  |
| **PREV CLASS**   **NEXT CLASS** | **FRAMES**    **NO FRAMES**     **All Classes** |  |
| SUMMARY: NESTED \| FIELD \| CONSTR \| METHOD | DETAIL: FIELD \| CONSTR \| METHOD |  |


---


## weka.classifiers.rules.part Class ClassifierDecList

```
java.lang.Object
  weka.classifiers.rules.part.ClassifierDecList
```

**All Implemented Interfaces:**: java.io.Serializable, RevisionHandler

**Direct Known Subclasses:**: C45PruneableDecList, PruneableDecList

---

``` public class ClassifierDecList extends java.lang.Object implements java.io.Serializable, RevisionHandler ```

Class for handling a rule (partial tree) for a decision list.

**Version:**
:   $Revision: 1.13 $

**Author:**
:   Eibe Frank (eibe@cs.waikato.ac.nz)

**See Also:**: Serialized Form

---

**Constructor Summary**

`ClassifierDecList(ModelSelection toSelectLocModel,
int minNum)`
  
          Constructor - just calls constructor of class DecList.


| **Method Summary** | |
| --- | --- |
| `void` | `buildDecList(Instances data, boolean leaf)`             Builds the partial tree without hold out set. |
| `void` | `buildRule(Instances data)`             Method for building a pruned partial tree. |
| `int` | `chooseIndex()`             Method for choosing a subset to expand. |
| `int` | `chooseLastIndex()`             Choose last index (ie. |
| `double` | `classifyInstance(Instance instance)`             Classifies an instance. |
| `void` | `cleanup(Instances justHeaderInfo)`             Cleanup in order to save memory. |
| `double[]` | `distributionForInstance(Instance instance)`             Returns class probabilities for a weighted instance. |
| `java.lang.String` | `getRevision()`             Returns the revision string. |
| `java.lang.String` | `toString()`             Prints rules. |
| `double` | `weight(Instance instance)`             Returns the weight a rule assigns to an instance. |

**Methods inherited from class java.lang.Object**

`equals, getClass, hashCode, notify, notifyAll, wait, wait, wait`

**Constructor Detail**

### ClassifierDecList

```
public ClassifierDecList(ModelSelection toSelectLocModel,
                         int minNum)
```

:   Constructor - just calls constructor of class DecList.


**Method Detail**

### buildRule

```
public void buildRule(Instances data)
               throws java.lang.Exception
```

:   Method for building a pruned partial tree.

    :   **Throws:**: `java.lang.Exception` - if something goes wrong

---


### buildDecList

```
public void buildDecList(Instances data,
                         boolean leaf)
                  throws java.lang.Exception
```

:   Builds the partial tree without hold out set.

    :   **Throws:**: `java.lang.Exception` - if something goes wrong

---


### classifyInstance

```
public double classifyInstance(Instance instance)
                        throws java.lang.Exception
```

:   Classifies an instance.

    :   **Throws:**: `java.lang.Exception` - if something goes wrong

---


### distributionForInstance

```
public final double[] distributionForInstance(Instance instance)
                                       throws java.lang.Exception
```

:   Returns class probabilities for a weighted instance.

    :   **Throws:**: `java.lang.Exception` - if something goes wrong

---


### weight

```
public double weight(Instance instance)
              throws java.lang.Exception
```

:   Returns the weight a rule assigns to an instance.

    :   **Throws:**: `java.lang.Exception` - if something goes wrong

---


### cleanup

```
public final void cleanup(Instances justHeaderInfo)
```

:   Cleanup in order to save memory.

---


### toString

```
public java.lang.String toString()
```

:   Prints rules.

    :   **Overrides:**: `toString` in class `java.lang.Object`

---


### chooseIndex

```
public final int chooseIndex()
```

:   Method for choosing a subset to expand.

---


### chooseLastIndex

```
public final int chooseLastIndex()
```

:   Choose last index (ie. choose rule).

---


### getRevision

```
public java.lang.String getRevision()
```

:   Returns the revision string.

    :   **Specified by:**: `getRevision` in interface `RevisionHandler`
    :   **Returns:**: the revision


---


|  |  |  |
| --- | --- | --- |
| **Overview** \| **Package** \| **Class** \| **Tree** \| **Deprecated** \| **Index** \| **Help** \| **Weka's home** | |  |
| **PREV CLASS**   **NEXT CLASS** | **FRAMES**    **NO FRAMES**     **All Classes** |  |
| SUMMARY: NESTED \| FIELD \| CONSTR \| METHOD | DETAIL: FIELD \| CONSTR \| METHOD |  |


---
